# Supplementary material for: Estimation of Lassa fever incidence rates in West Africa: Development of a modeling framework to inform vaccine trial design
Source: PLoS Negl Trop Dis. 2025 Jul 29;19(7):e0012751. doi: 10.1371/journal.pntd.0012751 (PMC12324683; doi:10.1371/journal.pntd.0012751)
Supplement: S1 Text — (DOCX) [file pntd.0012751.s001.docx]

**Supplemental Material for Estimation of Lassa fever incidence rates in West Africa: development of a modeling framework to inform vaccine trial design**

Sean M. Moore^1,^*, Erica Rapheal^2^, Sandra Mendoza Guerrero^3,a#^, Natalie E. Dean^4^, Steven T. Stoddard^3,a#^

^1^ Department of Biological Sciences, University of Notre Dame, Notre Dame, Indiana, USA

^2^ Independent Consultant, Minneapolis, Minnesota, USA

^3^ Emergent BioSolutions, Inc., Gaithersburg, Maryland, USA

^4^ Department of Biostatistics & Bioinformatics, Emory Rollins School of Public Health, Atlanta, Georgia, USA

^a#^Current address: Bavarian Nordic, Inc., Durham, North Carolina, USA

*[smoore15@nd.edu](mailto:smoore15@nd.edu)

**S1 Text**

**Model Details**

1. *Estimating the Force of Infection from Serology Data*

For administrative units with serology data binned by age group, we calculated the likelihood of the FOI (λ) based on the number of individuals between ages *a_1_* and *a_2_* (the upper and lower bounds of the age group) who tested positive, *P(a_1:2_),* out of the total number sampled, *T(a_1:2_)*. The likelihood was calculated for each age between *a_1_* and *a_2_* assuming a binomial distribution, *P(a_1:2_) ~ Binomial(T(a_1:2_),p(a))* where *p(a)* is the probability that an individual was seropositive as determined by equation (2). We then took a weighted average of the likelihood for each age group, with the weighting determined by the proportion of the population in that administrative unit in each age within the age group. We calculated the likelihood of the overall FOI (λ*_i_*) in administrative unit *i* by summing the logs of the binomial probabilities of *P_i_(a_1:2_)* across all age groups in a given seroprevalence dataset. The likelihood of a given FOI (λ*_i_*) for each administrative unit *i* was calculated across a range of values between 10^-6^ and 10^1^. We then fit a gamma distribution using the *optim* function in R to estimate shape and scale parameters representing the mean and variance in FOI (λ*_i_*), and these parameters were used as inputs to the subsequent steps in the modeling framework.

1. *Estimating Country-specific Reporting Fractions*

For each administrative unit where the FOI was estimated from serological data in the previous step, we estimated the fraction of LF infections from 2010-2023 that went unreported based on the discrepancy between reported LF cases and deaths and the number of infections predicted by the FOI estimates from those sites under the three different seroreversion scenarios. We assume that all individuals within an administrative unit are at risk of LASV infection and that each individual has the potential to fall into one of three categories during the study period: an observed LF case, an observed LF death, or unobserved. The unobserved category includes both individuals who were not infected and individuals who had an unobserved LASV infection (either because it was an asymptomatic infection or was an LF case/death that was not reported). We therefore modeled the distribution of person-years from 2010-2023 across these three categories: observed deaths, D; observed cases, C; and the total unobserved person-years, N. For administrative unit *i*, the total unobserved person-years, *N_i_*, is calculated as *N_i_* = *Y_i_* – *C_i_* – *D_i_*, where *Y_i_* = Σ*Pop_i_* is the sum of the annual population sizes from 2010-2023. The probability that a person of age *a* in year *y* would die from LF and be reported as such was,

$$\Pr(reported death,a\left| \lambda_{i},U_{i},\rho_{D},\pi\right)=\left( 1-p\left( a \right) \right)\frac{\lambda}{\lambda+\pi}\left( 1-e^{-\left( \lambda+\pi\right)} \right)\left( 1-U_{i} \right)\rho_{D}, (A1)$$

where *U_i_* is the proportion of infections that are unobserved in location *i* and ρ_D_ is the proportion of observed infections that result in death. Equation A1 represents the probability that an individual was susceptible at time *y*, and subsequently becomes infected in year *y* (based on the FOI, $\lambda$) and experienced an infection that was both observed and resulted in death. The probability of a reported case was the same as equation (A1) but with ρ_D_ replaced by 1 − ρ_D_. The probability of an unobserved person-year, $\Pr(unreported person\sim year,a\left| \lambda_{i},U_{i},\pi\right)$, takes into account the multiple ways in which a person-year would not result in a reported death or case, including currently being immune due to a past infection, by not being infected during the study time period from 2010-2023, or by being infected in year *y* but not being reported. From *Pr(reported death)*, *Pr(reported case)*, and *Pr(unreported person-year)* we calculate the probability of *D_i_*, *C_i_*, and *N_i_* among *Y_i_* total person-years, Pr(*D_i_, C_i_, N_i_* |$\lambda_{i}$, $U_{i},\rho_{D}$), using a multinomial distribution. Given estimates of λ_i_ from step 1, we used this distribution to estimate the parameter *U_i_* for each *i,* and country-specific values for $\rho_{D}$. The log likelihood of *U_i_* and $\rho_{D}$ was calculated by summing the logs of the probabilities from Pr(*D_i_, C_i_, N_i_* |$\lambda_{i}$, $U_{i},\rho_{D}$) assuming noninformative priors between 0 and 1 for all *U_i_* and a beta-distributed prior for $\rho_{D}$ with shape parameters 2.05 and 6.85. The posterior distributions of the parameters were then sampled using the BayesianTools package in R.

To extrapolate reporting fractions beyond the few administrative units with both serological and case/death data, we fit a Dirichlet distribution to the posterior predictions of the proportions of infections that result in a reported death, a reported case, or an unreported infection. For each draw *j* from the posterior, the proportions of reported deaths, reported cases, and unreported infections were calculated respectively as $\left( 1-\bar{U}_{j} \right)\rho_{D,j}$, ( $1- \rho_{D,j}$), and $\bar{U}_{j}$; with $\bar{U}_{j}$ $\rho_{D,j}$ representing the country-specific averages across all sites *i* within a country for each draw *j* from the posterior distribution. The corresponding Dirichlet parameters associated with reported cases, reported deaths, and unreported infections were $\alpha_{D}$, $\alpha_{C}$, and $\alpha_{U}$. We estimated these Dirichlet parameters by maximum likelihood using the *optim* function in R, treating posterior predictions of the proportion of infections that result in a reported death, a reported case, or an unreported infection as data points drawn from the Dirichlet distribution being fitted.

1. *Estimating LASV spillover rates*

For each administrative unit we next estimated the total number of annual infections, *I_i_,* based on the reported LF cases and deaths from 2010-2023 along with the estimated reporting probabilities from the previous step. The likelihood of a given number infections, *I_i_,* in administrative unit *i* was calculated using the Dirichlet-multinomial probability of obtaining the reported deaths, *D_i_*, and reported cases, *C_i_*, and *I_i_ - D_i_ - C_i_* unobserved infections following *I_i_* draws of those categories according to Dirichlet-distributed probabilities with parameters α_D_, α_C_, and α_U_ from step 2:

$$L\left( I_{i} | C_{i}{,D}_{i},\alpha_{D},\alpha_{C},\alpha_{U} \right)=\Pr\left( D_{i},C_{i},I_{i}-D_{i}-C_{i} | I_{i},\alpha_{D},\alpha_{C},\alpha_{U} \right).$$

We normalized the likelihoods from this equation across all values of *I_i_* to obtain posterior probabilities of each *I_i_,* which we used to obtain a set of posterior samples of *I_i_* for each admin1 and admin2 unit.

1. *Projecting the Force of Infection from estimated LASV spillover rates*

For each administrative unit where LASV spillover infections were estimated from LF case/death data, we projected the underlying FOI that would correspond to the estimated infection rate. The projected FOI*_i_* for each administrative unit *i* was obtained by minimizing the difference between the number of infections, I*_i_,* estimated in the previous step and the expected number of infections arising from a given FOI in the reverse catalytic model from equation (2) using the *optim* function in R. This resulted in a posterior distribution of FOI_i_ for each admin1 and admin2 unit.

1. *Estimation of Population-level Infection Histories*

The FOI projections generated from serology and case data in step 4 were next used to simulate population-level infection histories for each admin1 and admin2 unit. For the FOI projections, we drew 1000 samples for each administrative unit from the posterior distribution using the estimated shape and scale parameters from the gamma distribution estimated in step 1. For each value FOI_i,j_, (where j=1000 is the sample set) we computed the proportion of the population in administrative unit *i* that had been infected by age *a* using the catalytic model in equation (1), and the proportion of the population seropositive at age *a* using the reverse catalytic model in equation (2).

1. *Estimating LASV Infection and LF Attack Rates*

The population-level infection histories and FOI estimates were then used to compute the expected number of infections in each administrative unit i. We examined several different scenarios regarding the risk of seropositive or seroreverted individuals becoming reinfected and developing LF as described in the *Methods* section of the main text. The expected annual number of infections in administrative unit *i* were calculated from the FOI*_i_* using the reverse catalytic model for each of the 18 different scenarios at both the admin1 and admin2 levels. The number of infections was multiplied by the symptomatic probability (20%) to obtain an estimate of the expected number of LF cases in each administrative unit.

*Modeling the Force of Infection from Covariate Data*

The estimation of LASV spillover rates and the projection of the FOI from these spillover rates was performed for all admin1 and admin2 administrative units within the study region. However, LF cases or deaths have been reported in <50% of administrative units within the region (Figure 1), and infection estimates for these administrative units without any reporting are contingent on our estimated underreporting probabilities. In addition, there is also a large degree of uncertainty in the true spillover rate in administrative units that have reported only a small number of LF cases due to the large proportion of asymptomatic infections and low reporting probabilities. Therefore, we used several statistical models to explore the relationships between our FOI_i_ estimates from step 4 and several key spatial covariates (S2 Table). Due to the large number of potential covariates relative to the number of administrative units, we took several steps to simplify the statistical models. First, we tried to reduce the number of variables in our regression models by performing principal component analyses for monthly precipitation, monthly temperature, and monthly NDVI using the ‘prcomp’ function in R. The first two principal components (PCs) for NDVI explained >90% of the variation in monthly NDVI, and the first three PCs for precipitation and temperature each explained >90% of the variation for these two variables. We also tried performing a separate PCA combining all monthly climate variables to even further reduce the number of covariates. We next checked for multicollinearity in the linear regression model by examining variance inflation factors (VIFs) using the ‘CAR’ package in R. Latitude, as well as all of the climate-based PCs, had VIFs > 5 and were therefore excluded as covariates.

The statistical models were fit to the projected FOI_i_ estimates from administrative units with either serology data or reported LF case/death data (N=77 of 164 admin1s, N=372 of 1375 admin2s). The fitted models were then used to predict FOI in the administrative units with no serology or case data. Given that we have a relatively small dataset and high uncertainty in our projected FOI estimates, we considered eight different models, as well as a null model with a single FOI estimated across all administrative units. The first two models were linear regression models with or without two-way interaction terms between covariates. We also considered four different Gaussian Markov random field (GMRF) models: two with no covariates and two with linear effects of the covariates. GMRF models use a minimum mean squared error linear prediction with spatial covariance for spatial prediction or interpolation^1^. The GMRF models with or without linear effects for the covariates were run at two spatial resolutions: a low resolution (k=10 free parameters for admin1 and k=25 for admin2) or high resolution (k=40 for admin1 and k=100 for admin2). The seventh model was a random forest model implemented in R with the *randomForest* package. The eighth, and final, model was a boosted regression model implemented in R with the *gbm* package.

For each of the models we took 1000 samples from the posterior distributions of FOI*_i_* from step 4 and regressed log_10_ FOI against the covariates (where applicable), resulting in 1000 separate fits per model. The predicted values for both the fitted and unfitted administrative units from each model were then treated as point estimates to construct a set of 1000 posterior samples of FOI*_i_* for each administrative unit and each model.

*Ensemble model*

For each seroreversion scenario, we generated an ensemble model projection of FOI in each admin1 or admin2 using a form of stacked generalization^2^. Stacked generalization treats the eight statistical models as being at one level and uses the first-level models to generate a higher-order model that weights the predictions of the first-level models into its own prediction. The performance of each first-level model was assessed using a ten-fold cross-validation technique, with 90% of the data partitioned into the training set to fit the model, and the remaining 10% withheld for model validation. This process was performed ten times, with a different 10% of the dataset withheld for validation each time. Model performance was then assessed based on the model predictions on the withheld data and ensemble weights for each model were calculated based on relative model performance.

**Supplemental Results**

1. **Statistical and ensemble model results**

At the admin1 level, the most important covariates in the random forest model were longitude, travel time to the nearest urban center, and the Healthcare access and quality index (HAQ) (S3 Table). The most influential covariates in the boosted regression model were longitude, travel time to the nearest urban center, and the occurrence of *Mastomys natalensis* (S4 Table). At the admin2 level, the most important covariates in the random forest model were longitude, HAQ, and the extent of forest loss since 2000 (S5 Table).

*Ensemble model*

Our ensemble model consisted of a weighted combination of the FOI predictions of each individual statistical regression model, along with a noise term. Each model weight was calculated based on its negative marginal log likelihood in ten-fold cross-validation. Assuming a 6% seroreversion rate, the highest model weight for the admin1 ensemble model was the null model (43.4%), followed by the random forest model (22.9%), the low resolution GMRF without covariates (20.6%), and the linear model with interactions (7.8%). For the admin2 ensemble model the model weights of at least 5% in decreasing order were: the null model (53.8%), the boosted regression model (15.8%), the high resolution GMRF with covariates (11.6%), the linear model without interactions (7.0%) and the low resolution GMRF without covariates (6.1%). Due to the relatively strong weighting of the null model for both the 1^st^ and 2^nd^ administrative analyses, there is limited spatial heterogeneity in the FOI predictions from the ensemble model (Figure S7).

## **References**

1. Lindgren F, Rue H, Lindström J. An explicit link between Gaussian fields and Gaussian Markov random fields: the stochastic partial differential equation approach. *Journal of the Royal Statistical Society: Series B (Statistical Methodology)*. 2011;73(4):423-498. doi:10.1111/j.1467-9868.2011.00777.x

2. Bhatt S, Cameron E, Flaxman SR, Weiss DJ, Smith DL, Gething PW. Improved prediction accuracy for disease risk mapping using Gaussian process stacked generalization. *Journal of The Royal Society Interface*. 2017;14(134):20170520. doi:10.1098/rsif.2017.0520
