# Supplementary figures and images for: Estimation of Lassa fever incidence rates in West Africa: Development of a modeling framework to inform vaccine trial design
Source: PLoS Negl Trop Dis. 2025 Jul 29;19(7):e0012751. doi: 10.1371/journal.pntd.0012751 (PMC12324683; doi:10.1371/journal.pntd.0012751)

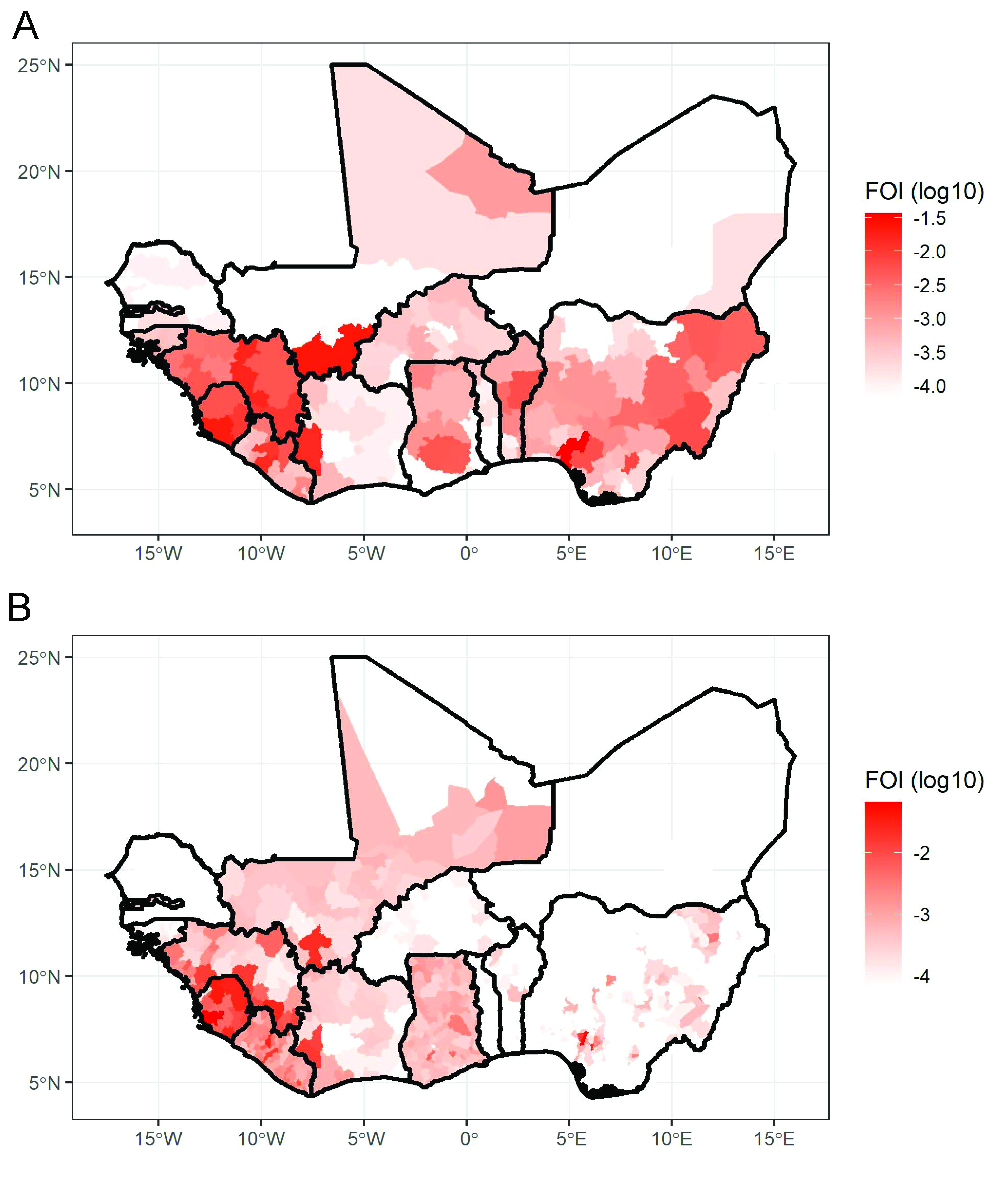

Supplement: S1 Fig — Maps of FOI projections from LF case/death data and reporting probabilities at the (A) 1st and (B) 2nd administrative levels with seroreversion = 0%. The base map layer was generated using GADM 3.6 data files which can be accessed from https://gadm.org/download_world36.html (TIF) [file pntd.0012751.s011.tif]

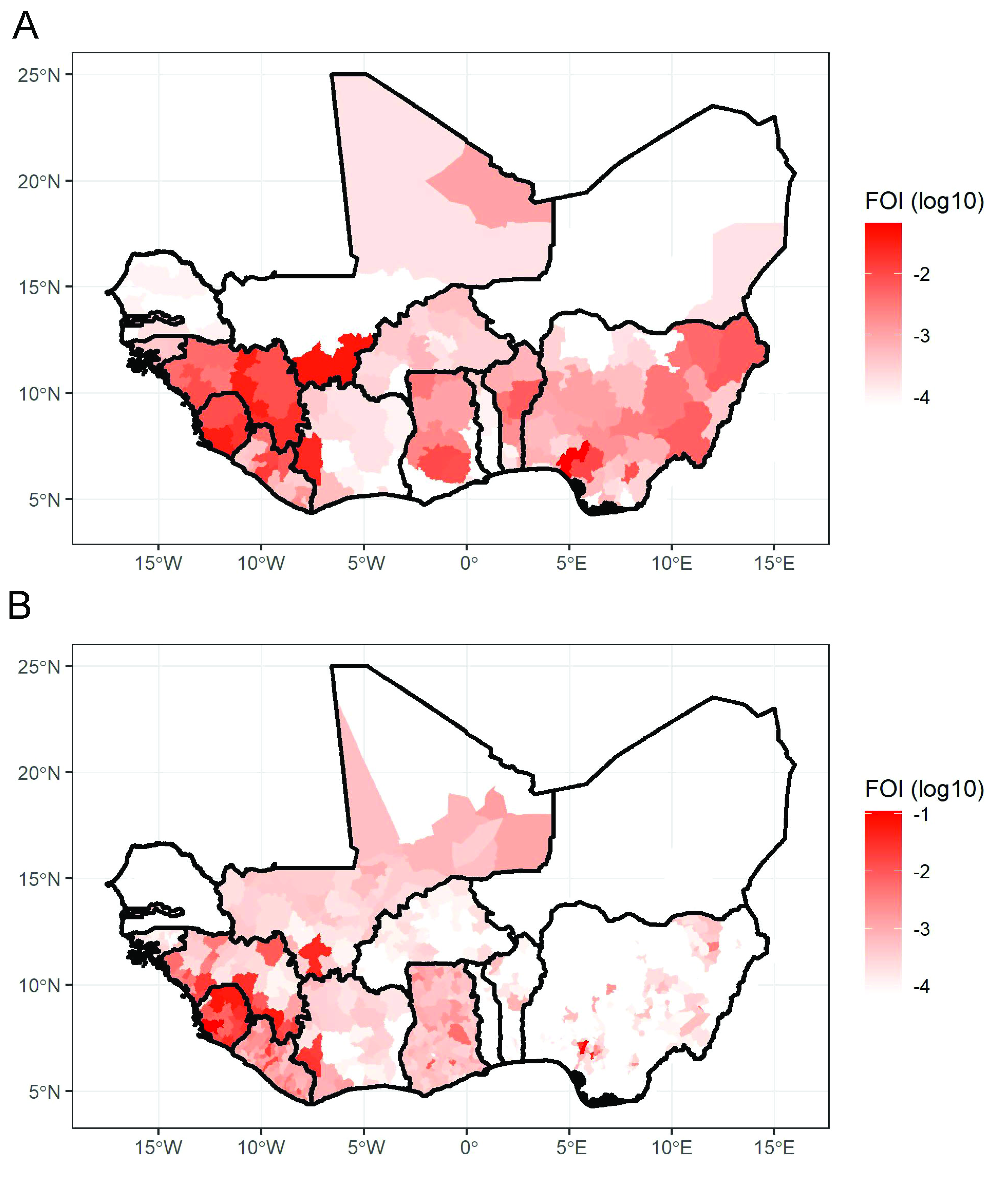

Supplement: S2 Fig — Maps of FOI projections from LF case/death data and reporting probabilities at the (A) 1st and (B) 2nd administrative levels with seroreversion = 3%. The base map layer was generated using GADM 3.6 data files which can be accessed from https://gadm.org/download_world36.html (TIF) [file pntd.0012751.s012.tif]

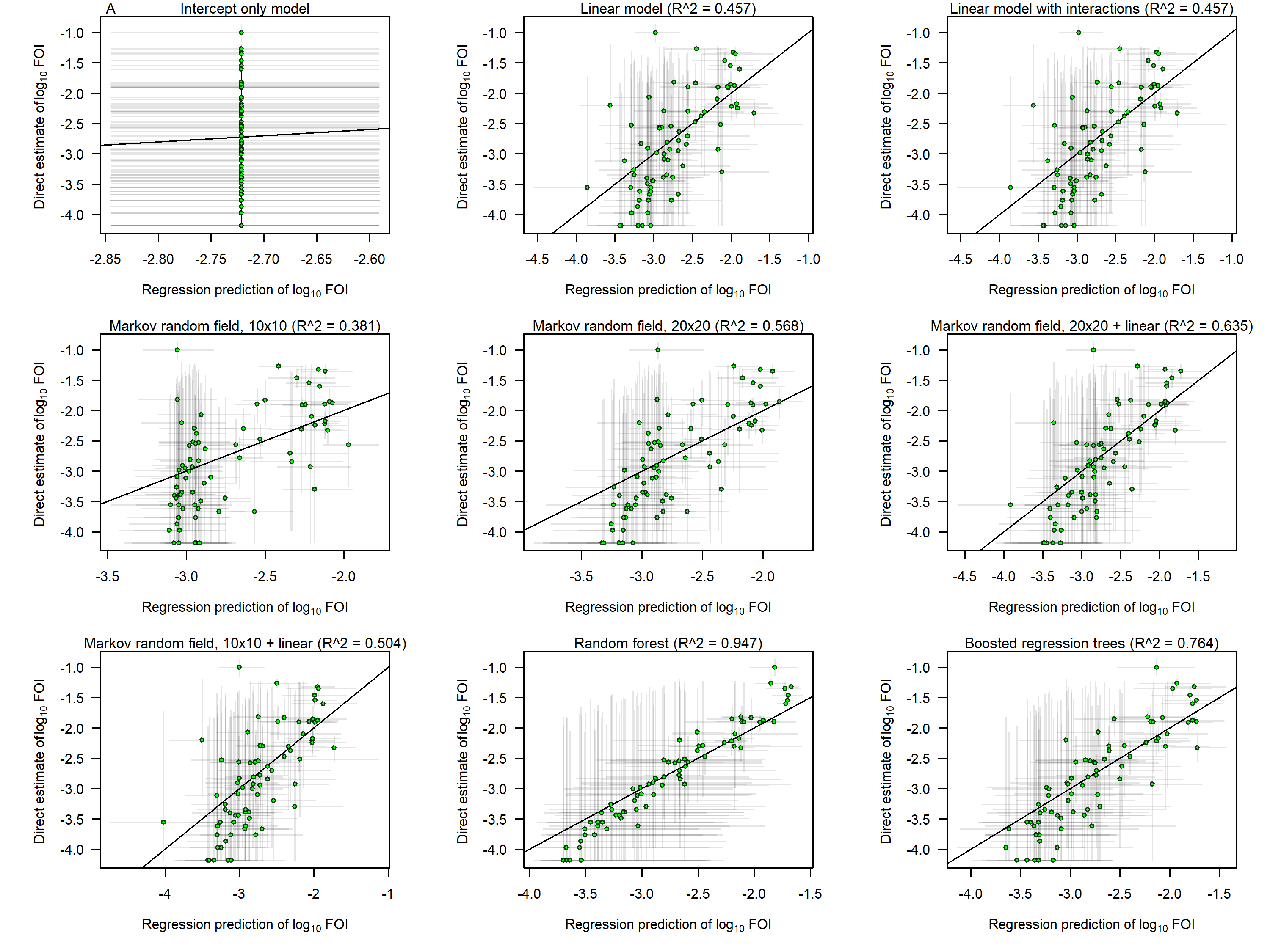

Supplement: S3 Fig — (TIFF) [file pntd.0012751.s013.tiff]

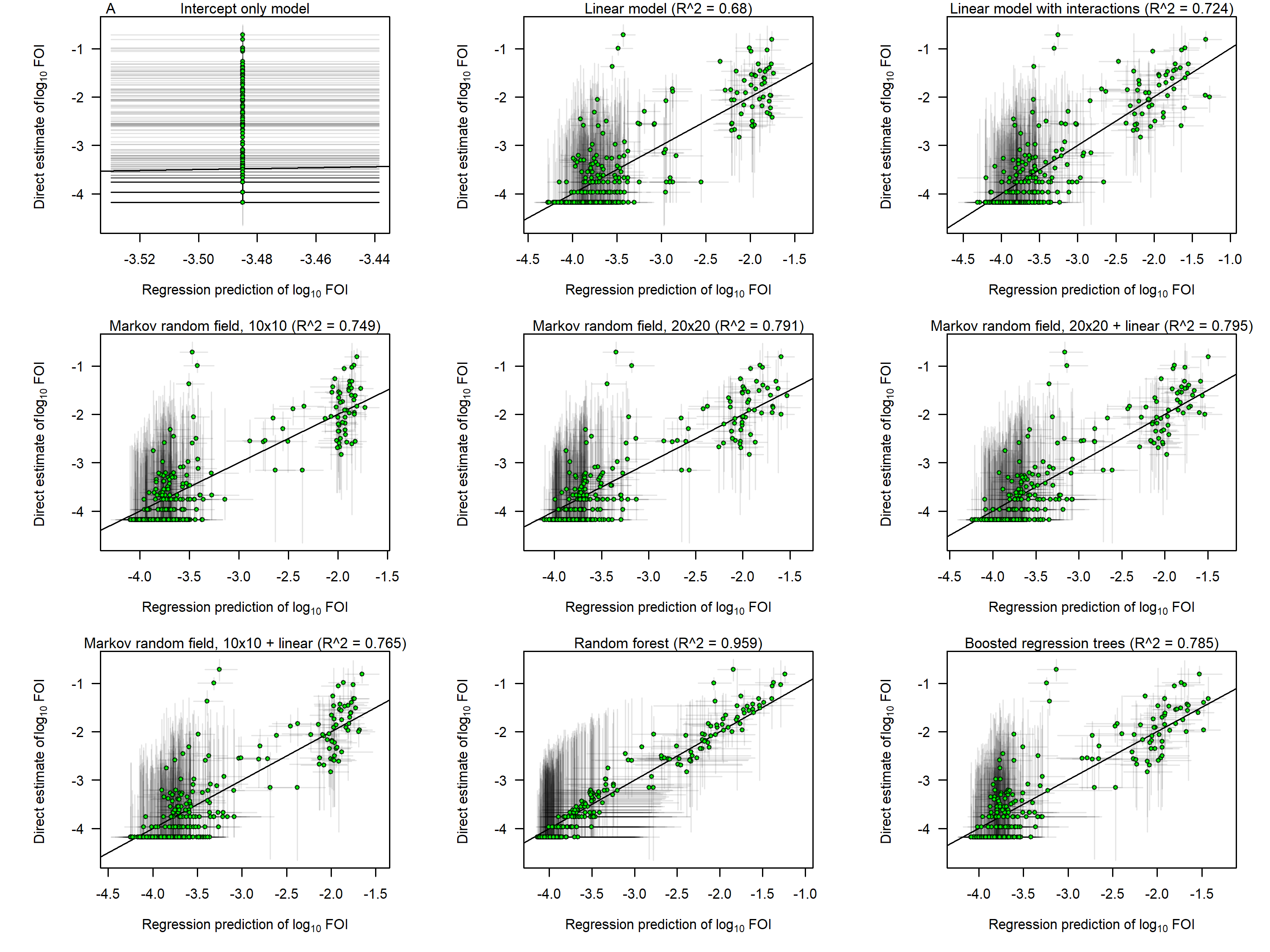

Supplement: S4 Fig — (TIFF) [file pntd.0012751.s014.tiff]

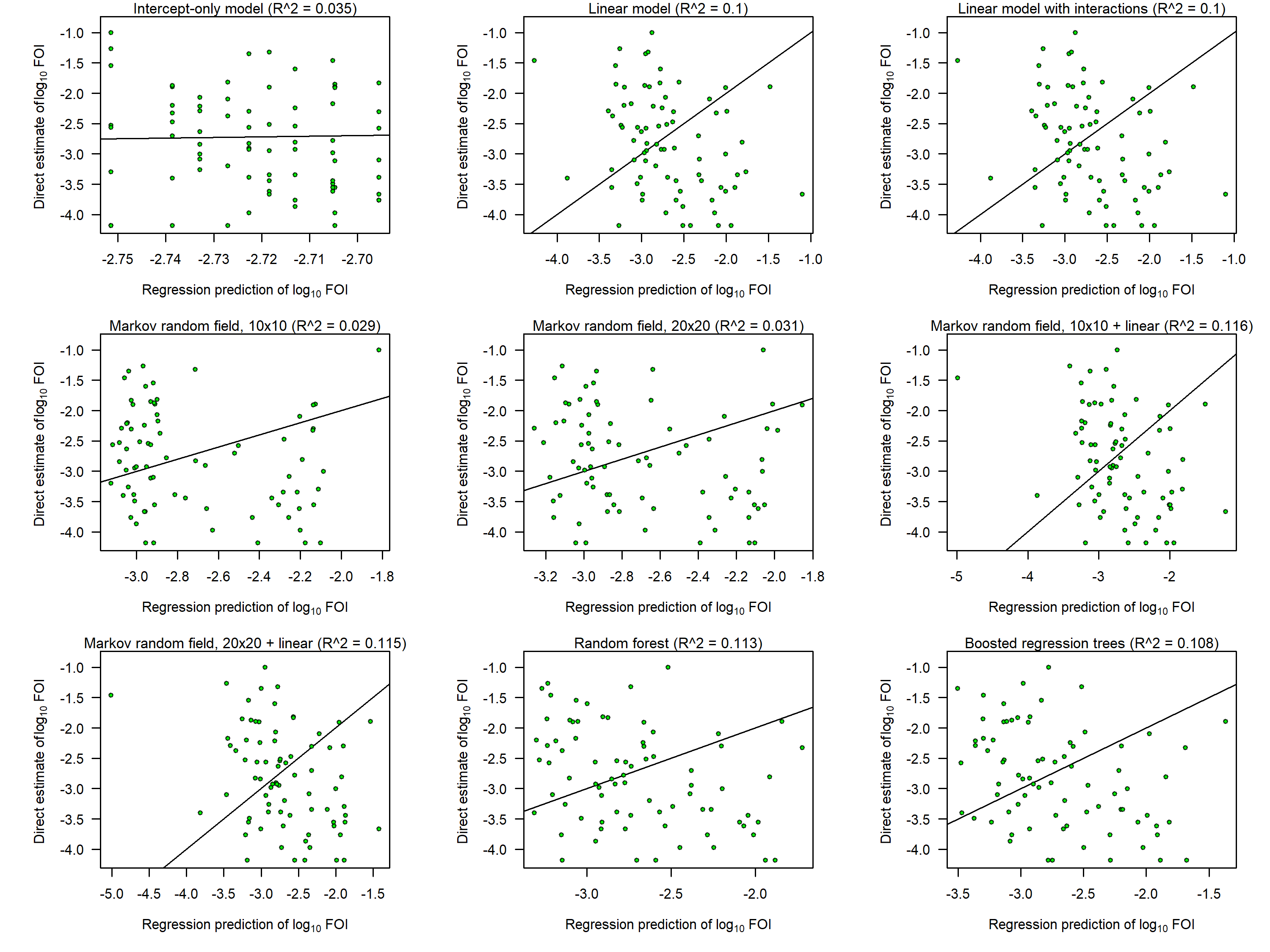

Supplement: S5 Fig — (TIFF) [file pntd.0012751.s015.tiff]

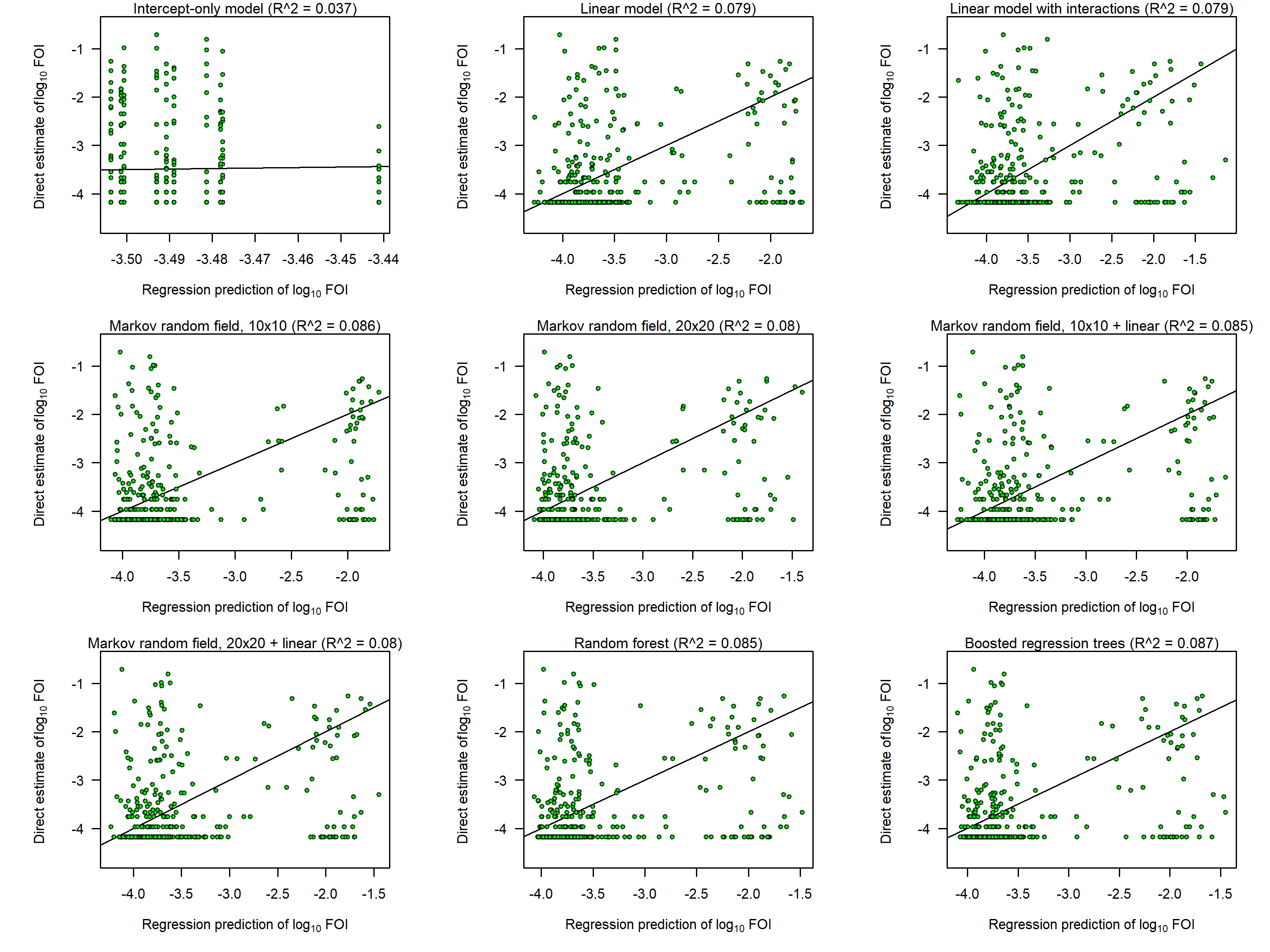

Supplement: S6 Fig — (TIFF) [file pntd.0012751.s016.tiff]

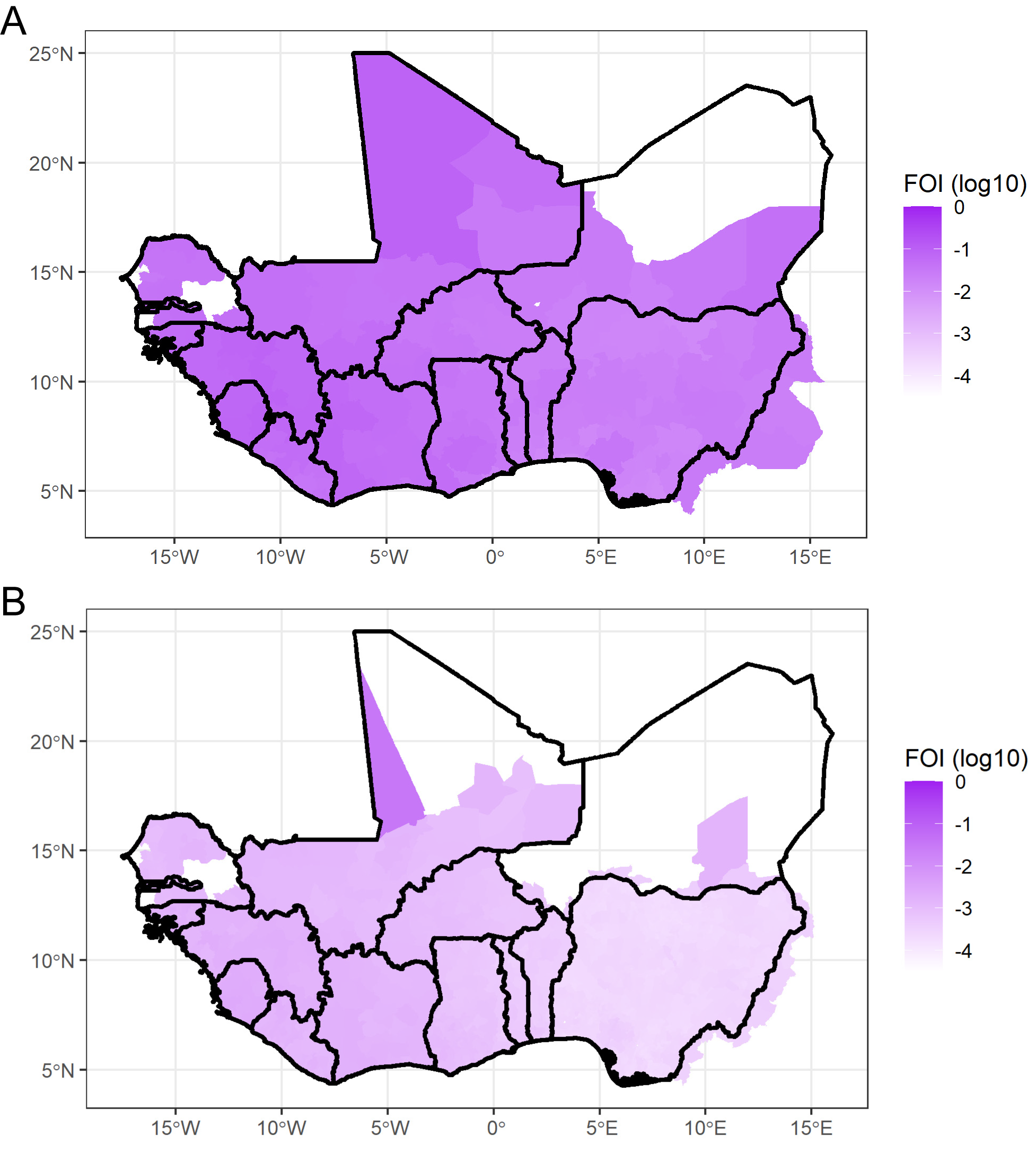

Supplement: S7 Fig — The base map layer was generated using GADM 3.6 data files which can be accessed from https://gadm.org/download_world36.html (TIF) [file pntd.0012751.s017.tif]

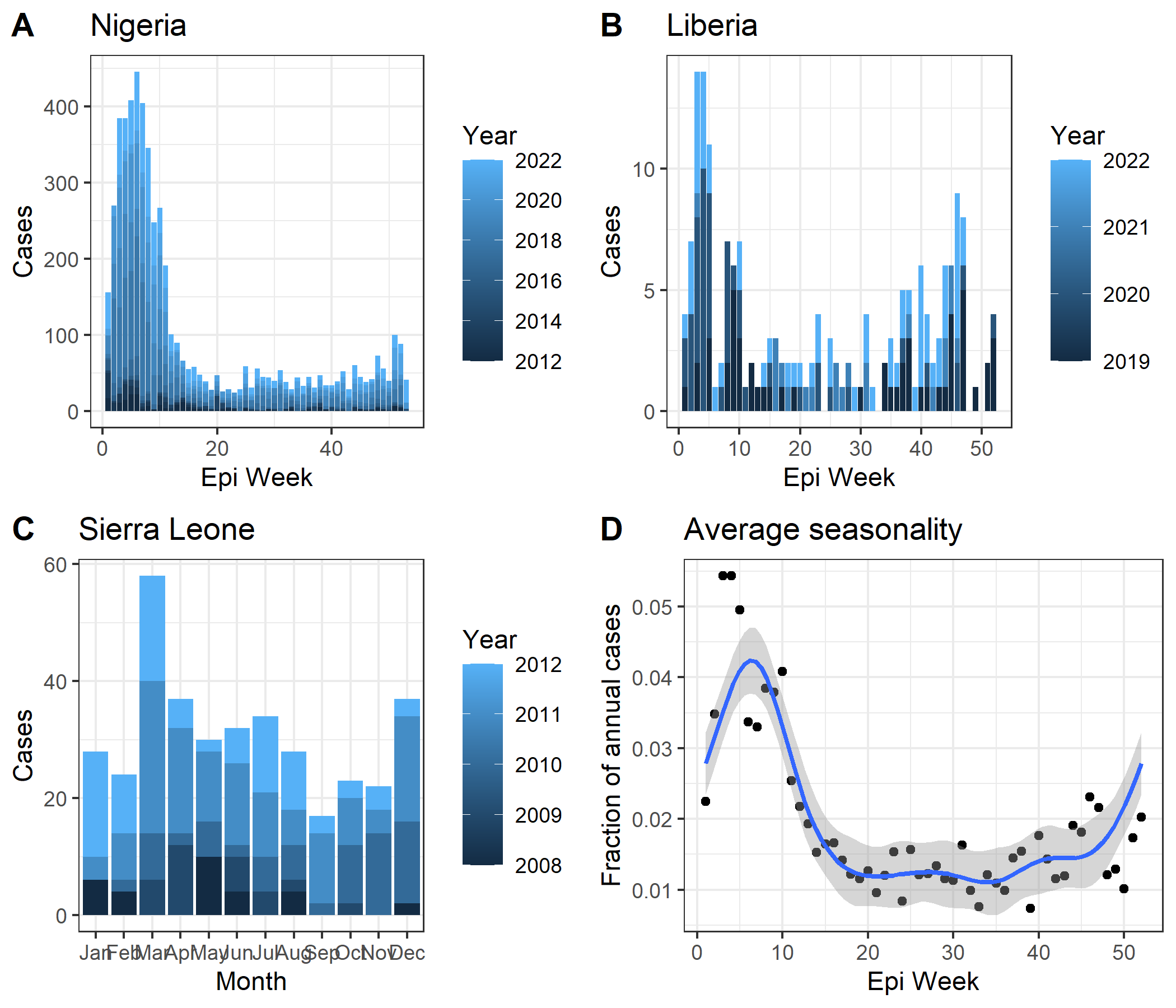

Supplement: S8 Fig — (TIF) [file pntd.0012751.s018.tif]
