# Supplementary material for: Estimation of Lassa fever incidence rates in West Africa: Development of a modeling framework to inform vaccine trial design
Source: PLoS Negl Trop Dis. 2025 Jul 29;19(7):e0012751. doi: 10.1371/journal.pntd.0012751 (PMC12324683; doi:10.1371/journal.pntd.0012751)
Supplement: Attachment — Submitted filename: Lassa_reviews.docx [file pntd.0012751.s020.docx]

**Response to Reviewers' comments**

Dear editor and reviewers,

Thank you for the thorough review and constructive comments to our manuscript entitled “Estimation of Lassa fever incidence rates in West Africa: development of a modeling framework to inform vaccine trial design”. We believe that we have addressed each of your comments and suggestions in this revised manuscript. Our responses to each comment are provided in italics after the comment.

Sincerely,

Sean Moore on behalf of all authors

Reviewer's Responses to Questions

**Methods**

-Are the objectives of the study clearly articulated with a clear testable hypothesis stated?

-Is the study design appropriate to address the stated objectives?

-Is the population clearly described and appropriate for the hypothesis being tested?

-Is the sample size sufficient to ensure adequate power to address the hypothesis being tested?

-Were correct statistical analysis used to support conclusions?

-Are there concerns about ethical or regulatory requirements being met?

Reviewer #1: This is a very important and relevant Manuscript especially with the prioritization of Lassa as a disease needing Countermeasures. The objectives are clear, smart and addresses a critical issue in Lassa research. The number of countries and sub nationals included for the modelling are quite appropriate. The Statistical analysis used to support the conclusions are also appropriate

*Thank you.*

Reviewer #2: The objectives of the study were clearly articulated and the study design was appropriate. To the best of my knowledge, the correct statistical analyses were used.

*Thank you.*

Reviewer #3: The manuscript frequently highlights substantial differences in observed seroprevalence at the village level, even within the same administrative level 2 region. This suggests that aggregating FOI at the chosen level of analysis may overlook critical heterogeneities. While the framing of the study as identifying potential vaccine trial sites at administrative levels 1 or 2 partly justifies this choice, the rationale for selecting this level of aggregation could be made more explicit. Specifically, the manuscript would benefit from a discussion of why a pixel-based approach was not used. Such an approach might better capture intra-regional variability, reduce the impact of administrative unit size on results, and more effectively represent heterogeneity in the included covariates. I am not asking for the analysis to be refactored at this level but I do believe a discussion of consideration of this would be suitable.

*We agree that a pixel-based approach would have been ideal to capture fine-scale heterogeneity in LASV spillover and to identify hotspots of transmission. However, our model relies on case data to infer reporting rates and then extrapolate FOI to areas without serological data and we do not have any fine-scale case data. In addition, we also only had 53 total seroprevalence datasets across 14 countries, which we decided was too few to make fine-scale estimates of spillover rates. We have added the following explanation to lines 264-269 (of marked up version) of the Methods section: “Although there is likely to be extensive spatial heterogeneity in LF incidence within an admin2 area (down to the village or sub-village level), case and death reports from national surveillance systems are typically aggregated to the admin1 or admin2 level. In the absence of finer scale case data, we were not able to estimate LF incidence at fine spatial scales using geostatistical modeling methods. There were also too few serological studies to reliably interpolate LASV spillover rates to finer spatial scales.”*

See summary and general comments for minor revisions suggested for the methods.

**Results**

-Does the analysis presented match the analysis plan?

-Are the results clearly and completely presented?

-Are the figures (Tables, Images) of sufficient quality for clarity?

Reviewer #1: The Analysis presented are satisfactory and match the plan. The results are clearly and completely presented. I find it important that the Authors understand that seroreversion does not necessarily equate to loss of protective immunity.

*Thank you.*

Reviewer #2: The results were clearly presented and consistent with the stated analytical plan. Figures and tables were excellent.

*Thank you.*

Reviewer #3: See summary and general comments for minor revisions suggested for the results.

**Conclusions**

-Are the conclusions supported by the data presented?

-Are the limitations of analysis clearly described?

-Do the authors discuss how these data can be helpful to advance our understanding of the topic under study?

-Is public health relevance addressed?

Reviewer #1: The conclusions are well made and supported by the data provided. The Authors recognize and have clearly stated the study limitation. These are due to many uncertainties about Lassa virus epidemiology

Reviewer #2: The conclusions were appropriate. Limitations could be enhanced (see below).

Reviewer #3: See summary and general comments for minor revisions suggested for the discussion/conclusion.

**Editorial and Data Presentation Modifications?**

Use this section for editorial suggestions as well as relatively minor modifications of existing data that would enhance clarity. If the only modifications needed are minor and/or editorial, you may wish to recommend “Minor Revision” or “Accept”.

Reviewer #1: ACCEPT

Reviewer #2: See comments below.

Reviewer #3: See summary and general comments.

**Summary and General Comments**

Use this section to provide overall comments, discuss strengths/weaknesses of the study, novelty, significance, general execution and scholarship. You may also include additional comments for the author, including concerns about dual publication, research ethics, or publication ethics. If requesting major revision, please articulate the new experiments that are needed.

Reviewer #1: This is a very strong study that will not only contribute to knowledge but will likely have immediate applicability in Clinical trial design for Lassa vaccine and Lassa therapeutic trials.

*Thank you for your comments.*

Reviewer #2: In their manuscript entitled, “Estimation of Lassa Fever Incidence Rates in West Africa: Development of a Modeling Framework to Inform Vaccine Trial Design”, Moore and colleagues determined the estimated force of infection of Lassa fever in administrative units in West Africa using previously published seroprevalence data as well as LF case and death reports. 31 published manuscripts were selected for review. Based on available serologic data, they were able to calculate the FOI for 24 1st level administrative units (Provinces/States) and 53 2nd administrative units (chiefdoms/municipalities). Assuming no seroreversion, Ondo State in Nigeria had the highest FOI at the Admin1 level and Moyamba District, Sierra Leone had the greatest FOI at the admin2 level. When seroreversion was accounted for, Ondo State still had the highest Admin1 FOI while OseLGA, Nigeria had the highest Admin2 rate. The random forest model proved to have the best fit among the statistical and machine learning models tested. Here, at the admin1 level, the most important covariates were longitude, travel time to nearest urban center and the HAQ. At the admin2 level, the most important covariates were longitude, HAQ and precipitation.

Lassa fever remains a significant health burden in West Africa, especially in the countries of Guinea, Sierra Leone, Liberia and Nigeria. There is increasing concern about cases appearing in other countries such as Benin and Togo. No vaccine is currently available, but several are in phase 1 trials and phase 2 trials are scheduled to begin soon. As the authors note, a greater understanding of the epidemiology of Lassa fever is needed to identify areas of West Africa that are best suited for vaccine trials. The design and conduct of these trials is complicated by the fact that an estimated 20% of infections result in disease severe enough to be recognized. Therefore, it has been estimated that a minimum annual incidence rate of 1% is needed to adequately power a vaccine efficacy trial. Unfortunately, there is a lack of prospective epidemiologic studies. In addition, there are several other factors which make determining the FOI accurately. First, and most importantly, this study is based on previously published seroprevalence studies that used very different diagnostic testing to determine sero-positivity. These differences make it very challenging to compare studies. Second, sero-reversion has been noted in many incidence studies. The authors accounted for these issues and others to develop 18 different models. Although I am not a statistician and their methods are beyond my area of expertise, the approach seems very thorough.

Overall, this is a well written manuscript that will increase our understanding of Lassa virus epidemiology. In addition, the results challenge commonly held notions about LF incidence. For example, in Sierra Leone, the districts of Kenema and Kailahun have traditionally been considered LF hyperendemic areas. The results described in this manuscript challenge these beliefs and may result in improved design of vaccine trials and the identification of areas most at risk and, consequently, the populations that would most benefit from a vaccine.

Specific Comments:
1. Line 396: This line has an incomplete sentence that must be revised.

*This sentence has been fixed and now states: “The most influential covariates in the boosted regression model were longitude, travel time to the nearest urban center, and the estimated probability of LASV presence in Mastomys natalensis (Table S4).”*

2. Discussion: The section on limitations neglects several key factors. First, it is certainly possible that LF epidemiology has changed over the past 20 years. Including all studies equally may bias the results. Second, virtually all studies used a different platform for determining seropositivity. They may not be comparable. Third, Lassa virus is now considered to be made up of as many as 7 different lineages. It is conceivable that not all lineages are equally likely to cause infection.

*We have added the following to the Limitations subsection to acknowledge these issues:*

*“The epidemiology of LF may have also changed over the course of the period covered in our datasets, which might bias our results if spillover rates have significantly increased or decreased in some regions. We assumed a constant rodent-to-human FOI because we did not have sufficient data to detect temporal shifts in FOI. The case and death data contained limited phylogenetic information, such as the LASV lineage, that might affect disease severity, and therefore the probability of a case being detected. There is also variability in the sensitivity and specificity of the different serological tests used in the studies included in our analysis, as well as variability in the LF case definitions used in different countries, which could lead to temporal or spatial biases in case surveillance.”*

Reviewer #3: Review of - Estimation of Lassa fever incidence rates in West Africa: development of a modeling framework to inform vaccine trial design
Thank you for the opportunity to review this manuscript which investigates the force of infection (FOI) of Lassa virus (LASV) in endemic regions of West Africa. The authors aim to model the spatially explicit FOI across West Africa using serological survey data and published case data. The manuscript is generally well-written, with a comprehensive description of methods in both the main text and is expanded upon in supplementary materials. The motivation behind the study is clear, and the results are both interesting and contextualized effectively within the current limitations of our understanding of LASV transmission in endemic regions.

The authors are transparent about the key assumptions underpinning their modeling and interpretation of results. However, the literature review component of the manuscript could be better described, and its role in identifying key covariates and parameter estimates should be more explicitly justified.

*We have added an additional subsection “Literature review and epidemiological data” to the beginning of the Methods section at line 153.*

While there are strong assumptions associated with the chosen methods, which may not fully reflect the complexity of LASV transmission, the analysis is robust and provides an excellent foundation for refinement as higher-quality observational data become available. This work will undoubtedly serve as a valuable resource within the scientific literature on Lassa fever. I recommend the manuscript for publication but suggest addressing the following comments to strengthen the study further.

I have attempted to run the code provided in the linked github repository but ran into some difficulties as a file required for analysis has not be provided (the priors for step2_ results/prior_iceberg.RData). The authors have made the underlying data available.

*The github repository has been updated and the code to replicate our analysis should now be runnable with the required R packages.*

Major comment
The manuscript frequently highlights substantial differences in observed seroprevalence at the village level, even within the same administrative level 2 region. This suggests that aggregating FOI at the chosen level of analysis may overlook critical heterogeneities. While the framing of the study as identifying potential vaccine trial sites at administrative levels 1 or 2 partly justifies this choice, the rationale for selecting this level of aggregation could be made more explicit. Specifically, the manuscript would benefit from a discussion of why a pixel-based approach was not used. Such an approach might better capture intra-regional variability, reduce the impact of administrative unit size on results, and more effectively represent heterogeneity in the included covariates. I am not asking for the analysis to be refactored at this level but I do believe a discussion of consideration of this would be suitable.

*As described above in the Methods section of the review, we have added a justification to our approach on lines 264-269 of the Methods section*

Minor comments

Abstract:

Line 25. You estimate the FOI of LASV in human populations driven by transmission from the zoonotic host (M. natalensis, but potentially other rodent and shrew species). Should this more explicitly be termed the force of infection from rodents to humans? Rodent-to-rodent transmission (and therefore a rodent level FOI) occurs in the same settings and is likely important to the human risk of infection but is not modelled in your approach. Similarly the role of human-to-human transmission is likely underestimated in community settings and not explicitly modelled in your approach. There is also increasingly discussion of reverse zoonosis (human-to-rodent FOI), particularly based on studies conducted in Guinea.

*Edited this sentence to “… we estimated the force of infection (FOI) from rodent hosts to humans in all 1^st^ and 2^nd^ administrative units…”*

Author summary:

Line 43. Replace the oft-cited estimates from reference 3 with the more recently modeled estimates in reference 25. While you critique reference 25 in your discussion, its methodology is more transparent than that of reference 3, which lacks any detail on how its estimates were derived.

*We have updated the estimate here to the central estimates (with and without reinfection) for Basinski et al. [ref 25 in original MS]. In the introduction we provide a bit more context regarding this and prior infection estimates (see response to comment below).*

*“Lassa fever virus infects an estimated 0.9 to 4.3 million people and kills thousands annually in West Africa.”*

Introduction:

Line 60. Again this statement is derived from reference 3. The 20% is based on 48 known infections of which 9 were identified as ill. This is not equivalent to severe, life-threatening hemorrhagic illness and it may be more suitable to report this as “up to 20% of infections are thought to result in symptoms ranging from mild (i.e., headache, fever) through to severe (i.e., coma, acute renal failure) or fatal hemorrhagic illness”. There are several publications from the NCDC where they report disease outcomes (for example, <https://pmc.ncbi.nlm.nih.gov/articles/PMC6537738/>). While you highlight this further in Line 127-129 it may be worth moving this to this section.

*We have edited this sentence to read “Most LASV infections are presumed to be asymptomatic or sub-clinical, but up to 20% of infections are thought to result in symptoms ranging from mild (i.e., fever, headache) through to severe (i.e., coma, acute renal failure) or fatal hemorrhagic illness.2”*

Line 61. Provide a national-level case fatality rate (CFR) from a single year for better clarity. For instance, the NCDC reported a CFR of 17.9% in 2023 based on 1,270 confirmed cases from 28 states ([https://ncdc.gov.ng/ncdc.gov.ng/themes/common/files/sitreps/60b4a539bd9b9852ac1a5059ec0f3433.pdf](https://gcc02.safelinks.protection.outlook.com/?url=https%3A%2F%2Fncdc.gov.ng%2Fncdc.gov.ng%2Fthemes%2Fcommon%2Ffiles%2Fsitreps%2F60b4a539bd9b9852ac1a5059ec0f3433.pdf&data=05%7C02%7Csean.moore2%40nih.gov%7C312a86d2203b4e5acc3708dd407ae923%7C14b77578977342d58507251ca2dc2b06%7C0%7C0%7C638737619280749724%7CUnknown%7CTWFpbGZsb3d8eyJFbXB0eU1hcGkiOnRydWUsIlYiOiIwLjAuMDAwMCIsIlAiOiJXaW4zMiIsIkFOIjoiTWFpbCIsIldUIjoyfQ%3D%3D%7C0%7C%7C%7C&sdata=VuN8SHkaoLUXygubGz%2B%2BHF7KVcHjYtiLEVGxAE3H61k%3D&reserved=0)). As the CFR is highly dependent on case detection some states within Nigeria report CFR as 100%.

*We have added a reference to the 2022 and 2023 CFR in Nigeria, and clarified that CFR is dependent on case detection: “Case fatality rates (CFRs) among reported or hospitalized LF cases are dependent on the likelihood of case detection, but can reach 60% when detection of severe cases is delayed.^1^ In both 2022 and 2023, Nigeria reported a CFR of 18%, which indicates that the CFR remains high even after improvements to the disease surveillance and response systems.^3^”*

Line 62-65. See comment above about using updated estimates.

*We have added the updated estimates from Basinski et al. while also leaving in a mention of the older estimates, since those are still frequently cited: “Previous studies have estimated that hundreds of thousands—and potentially as many as 0.9 to 4.3 million—LASV infections and 5,000 to 18,000 LF deaths occur annually in sub-Saharan Africa.^4–6^ However, these estimates are highly uncertain as they were either extrapolated from limited serological studies conducted decades ago or generated from modeled spillover rates from the rodent reservoir to humans.”*

Line 77-78. I assume the description of an ideal study site is derived from CEPI material but it would appear to me that an ideal study site will also need an ongoing assessment of pathogen circulation in rodents to quantify risk of spillover into human populations. As it is unclear how the “presence of frequent LASV spillover” will otherwise be assessed.

*We agree that in addition to using human serology studies to assess recent transmission, it might also be important to establish that the pathogen is actively circulating in the local rodent population. We have edited the text as follows:” An ideal study site will have a minimum baseline seroprevalence level and/or evidence of ongoing circulation of the virus in the local rodent population as indicators of the presence of frequent LASV spillover. However, the baseline seroprevalence level must be low enough that existing immunity in the population does not substantially reduce the LF incidence rate.”*

Line 86-89. This is potentially true but this is unlikely the sole reason these cases are not detected. Access to healthcare in the locations in which LASV spillovers occur is highly variable across the region, with time varying components (e.g., difficulty in travel due to road conditions, availability to leave farming communities during harvesting periods etc.) or financial constraints (e.g., direct- and indirect costs of healthcare). It would be useful to include some non-clinical considerations in lack of case detection.

*We agree that clinical similarities are only one reason that LF cases go undetected, and that access to healthcare is also a key factor. We have edited this section and added a mention of varying HC access:*

*“A majority of LF cases are not detected by current clinical surveillance systems, at least in part because common LF symptoms—such as fever, malaise, headache, and muscle pain—closely resemble those of other febrile illnesses endemic to the region.^1,2,13^ Access to healthcare facilities varies considerably,^14^ which—along with other factors—influences the probability that symptomatic individuals will seek treatment.^15,16^”*

Line 113-116. Abundance of rodents and Infection prevalence in rodents is also temporally heterogeneous within small spatial scales (i.e., at village level) not only in a seasonal pattern across broad spatial scales. Further, rodent detection rates =/= rodent abundance which complicates our understanding of rodent ecology and pathogen prevalence within these settings. A detailed description of M. natalensis ecology and our current uncertainties in the effect of this on spillover rates is beyond the scope of this manuscript. However, a sentence around rodent ecology and the rodent-human contact nexus within these contexts would fit well with your mention of “individual and community-level risk factors for infection”.
*We have added a brief mention of how temporal patterns may be driven by rodent population dynamics and/or contact rates with humans: “Moreover, incidence of disease can be markedly seasonal, suggesting important variation in either rodent infection rates or contact with humans as a result of rodent population dynamics and movement patterns associated with rainfall and agricultural practices that affect food availability.^22,28^”*

Methods:
Epidemiological data:
For clarity it may make sense to have subheadings in this section for infection data and case data. This may also make it clearer later when referencing which dataset the reported model outcomes are based on.

*We have created separate subheadings for Serology and Case/death data in the Methods section.*

Line 161-169. It is unclear what the final epidemiological dataset that was used is. In S1 of reference 61 11 references are included (1 suggests NCDC situation report data with another being ProMED reports (which are no longer available)). You later report excluding all those conducted prior to 1980 (it is not reported why you have chosen this cutoff), which leaves 6 of the 11 references as potentially included. I believe the final dataset is this ([https://github.com/mooresea/lassa-model/blob/main/data/case_reports_Lassa_CASES.csv](https://gcc02.safelinks.protection.outlook.com/?url=https%3A%2F%2Fgithub.com%2Fmooresea%2Flassa-model%2Fblob%2Fmain%2Fdata%2Fcase_reports_Lassa_CASES.csv&data=05%7C02%7Csean.moore2%40nih.gov%7C312a86d2203b4e5acc3708dd407ae923%7C14b77578977342d58507251ca2dc2b06%7C0%7C0%7C638737619280766668%7CUnknown%7CTWFpbGZsb3d8eyJFbXB0eU1hcGkiOnRydWUsIlYiOiIwLjAuMDAwMCIsIlAiOiJXaW4zMiIsIkFOIjoiTWFpbCIsIldUIjoyfQ%3D%3D%7C0%7C%7C%7C&sdata=Q3bSAJy%2B7xYPPUB61%2Bl8eDLa2vBEwqvOnS2ZfzguEvM%3D&reserved=0)) for cases and ([https://github.com/mooresea/lassa-model/blob/main/data/case_reports_Lassa_IGG.csv](https://gcc02.safelinks.protection.outlook.com/?url=https%3A%2F%2Fgithub.com%2Fmooresea%2Flassa-model%2Fblob%2Fmain%2Fdata%2Fcase_reports_Lassa_IGG.csv&data=05%7C02%7Csean.moore2%40nih.gov%7C312a86d2203b4e5acc3708dd407ae923%7C14b77578977342d58507251ca2dc2b06%7C0%7C0%7C638737619280780794%7CUnknown%7CTWFpbGZsb3d8eyJFbXB0eU1hcGkiOnRydWUsIlYiOiIwLjAuMDAwMCIsIlAiOiJXaW4zMiIsIkFOIjoiTWFpbCIsIldUIjoyfQ%3D%3D%7C0%7C%7C%7C&sdata=WR2kjwAy5jksJ%2BfuMPX0PJ2QPN9kjN2RYfL4fgxXOUs%3D&reserved=0)) for infection, it may be worth directly referencing these in the text as readers may not go to the github repository otherwise. How did you de-duplicate data or decide on which values to use? For example the Redding dataset is derived from NCDC data which you use for previous years and in the past I have had difficulties reconciling case counts from Liberia across multiple publications.

*We only included a single data source for each administrative unit and year, whichever seemed most comprehensive based on either case count or the length of the observation. Regarding consolidating datasets, on review we discovered that we have double counted cases in a few countries when aggregating at the admin1 level if there were data sources for both the admin1 level and admin2 units within that admin1 unit. We have corrected this issue by removing admin2 observations when more complete admin1 observations are available and rerun all steps of the model to re-estimate LF incidence. Because the number of duplicates was limited, the results are qualitatively the same and only most incidence rates changed by 0.1 per 1000 or less (see Table 1).*

Clarify the reasoning for excluding studies prior to 1980 and whether this cutoff impacts representativeness.

*We have added the following text on lines 202-204 of the Methods section: “Seroprevalence studies prior to 1980 were excluded because there have been large shifts in both demographics and land usage within the study region the likely impact viral circulation within rodent populations and spillover to humans in the past 40 years.”*

Covariate data:
Did you assess collinearity among covariates to mitigate potential confounding effects? This may be particularly relevant for the travel-time-to-healthcare and land-use covariates. The statistical models are described in more depth in the supplementary material, it appears that a large number of potential covariates for limited observational data were used.

*Thank you for this comment. We did not initially test for multicollinearity. We have now done so by examining variance inflation factors and determined that latitude, as well as all of the potential climate variables (even when latitude is excluded), have VIFs > 5 indicating collinearity. We have removed these variables and rerun our models*. *Removing these variables did not lead to large changes in initial model fits (as measured by R^2), but did lead to a decline in performance in predicting the out-of-sample values (figures S5,S6), making the ensemble model predictions even more homogenous and less informative. We had already placed only a limited emphasis on the ensemble model predictions due to poor performance and in the update manuscript we have removed some additional details about spatial heterogeneity that are no longer relevant.*

Model:
Line 233-235. Is it reasonable to assume a constant FOI over time given fluctuations in rodent abundance and LASV prevalence within the rodent populations? I understand that a time-varying FOI may not be suitable given data resolution limitations and computational burdens but I think if this is a necessary simplifying assumption this should be expanded upon further in the methods section or discussion.

*Unfortunately, we did not have sufficient data, such as multiple serological datasets over time from the same location, to detect changes in FOI over time. We added the following sentence to the Limitations section of the Discussion: “We assumed a constant rodent-to-human FOI because we did not have sufficient data to detect temporal shifts in FOI.”*

Line 278-287. If I understand this correctly this section is about inferring the number of infections that would be symptomatic. Is it worth discussing that the ENABLE study presumably has intensive investigation of symptoms for individuals found to seroconvert/reporting of symptoms and subsequent testing and may not represent what might be considered a symptomatic disease in the absence of an intensive prospective cohort study, potentially overestimating the rate of symptomatic disease?

*We agree that the ENABLE study is likely to have a higher detection rate of mild or moderate LF cases than passive surveillance systems. However, we are using these estimates assuming a large-scale vaccine trial that would have a similar surveillance to the ENABLE study.*

Results:
Line 328-341. You introduce an in-depth review of the published literature without previously describing it in the methods. Is this section in reference to Lines 167-172 in the epidemiological data section? If so I think there needs to be a bit more information on how these were selected? Was this based on a systematic search and review of the literature or a review of author identified research? How did you assess whether this was a comprehensive set of manuscripts? It is unclear how studies of imported LF cases would be helpful in inferring FOI in the endemic region.

*We have added an additional subsection “Literature review and epidemiological data” to the beginning of the Methods section at line 153.*

Line 336-338. Studies from Guinea have fairly consistently shown higher prevalence of infection among rodent populations and consistent evidence of LASV prevalence. However, this may not be the case across the endemic region, it may be worth highlighting examples of the inverse, where LASV may not be circulating consistently (i.e., <https://pmc.ncbi.nlm.nih.gov/articles/PMC7824740/>).

*We added a sentence to mention that LASV is found at lower levels in some areas: “However, LASV also appears to circulate at lower levels in other endemic areas, such as Bo, Sierra Leone, where 2.8% of rodents tested positive over a two-year period from 2014-2016.*^63^*”*

Line 367-370. Clarify whether the "average" reported is the mean or median. A median may be more appropriate, given the likely non-normal distribution of probabilities.

*The values presented were median values, not mean values, so we have changed the wording to properly reflect this fact. There were slight changes to the reporting values at the admin1 level due to the fix regarding overlapping datasets described above (overall median reporting probability changed from 0.18% to 0.17%).*

Line 396-7. Incomplete sentence? “. center,”

*This sentence has been fixed and now states: “The most influential covariates in the boosted regression model were longitude, travel time to the nearest urban center, and the estimated probability of LASV presence in Mastomys natalensis (Table S4).”*

LASV infection attack rates and incidence and figure 6. I am a bit concerned about the age stratified results. Based on published data seroprevalence generally increases with age but the rate of increase varies with site. Despite this the proportion of cases that are detected in individuals under 18 is a minority of reported cases. To my knowledge there is not much information about the varying symptomatology by age but stratifying your results into <12, 12-17 and 18+ suggests that we’d be expecting most symptomatic disease to occur in <12 year olds. Can I suggest showing your results in a different way? Perhaps a figure of LASV incidence (infection) or LF incidence (disease) with age on the x-axis (continuous time or 5-10 year age bands) and annual incidence per 1,000 on the y-axis. The different sites can be represented by colours with associated credible/confidence intervals. Or perhaps panelling by sites if it becomes difficult to interpret overlain colours. In this way I think it would be easier to appreciate that the rate of infection or disease varies by time but there is a consistent pattern across some sites.

*We have updated figure 6 with Age on the X-axis as a continuous variable. It is now a 6-panel figure with each panel corresponding to one of the admin2 districts with the highest median incidence rate. Because there is not any good data on LF symptomology by age we have assumed that the probability of detecting an LASV infection is not age-dependent.*

Seasonality. The associated figure (fig 8.) could be moved to supplementary. It doesn't appear that this is a new analysis and contains a regression model fitted to reported case counts which have previously been discussed to represent substantial underreporting. The figure should be titled seasonality of LF case reporting rather than incidence. I don’t think much of the data is based on the date of symptom development of the identified cases and is instead the epi-week or month in which they have been added to the relevant line list. There is a wide range of incubation period and symptom onset reported in the literature ([https://doi.org/10.1016/S2214-109X(24)00379-6](https://gcc02.safelinks.protection.outlook.com/?url=https%3A%2F%2Fdoi.org%2F10.1016%2FS2214-109X(24)00379-6&data=05%7C02%7Csean.moore2%40nih.gov%7C312a86d2203b4e5acc3708dd407ae923%7C14b77578977342d58507251ca2dc2b06%7C0%7C0%7C638737619280793501%7CUnknown%7CTWFpbGZsb3d8eyJFbXB0eU1hcGkiOnRydWUsIlYiOiIwLjAuMDAwMCIsIlAiOiJXaW4zMiIsIkFOIjoiTWFpbCIsIldUIjoyfQ%3D%3D%7C0%7C%7C%7C&sdata=00LLzoHuGEgfwDo%2Blaj3aCG1YyRAe11bGVosDCa7B5c%3D&reserved=0)).

*We moved this figure to the supplement (Fig S8) and changed the title wording as requested.*

Discussion:
It may be worth including a line in the discussion around line 583-586 about the length of time vaccine trials may need to be completed over (i.e., years) to account for the seasonal dynamics expected in spillover events/human cases.

*We added a suggestion regarding the necessity of multi-year trials to line XXX: “ensuring sufficient statistical power may require a large increase in the number of individuals enrolled in a field trial or a longer trial period spanning multiple transmission seasons.”*

Not too sure if this is more relevant for the methods section or limitations within the discussion but case definitions over the study period will vary by country, time period and diagnostic test availability. Thus there may be a time varying surveillance bias/undercounting of cases.

*We have added the following sentence to the limitations section: “There is also variability in the sensitivity and specificity of the different serological tests used in the studies included in our analysis, as well as variability in the LF case definitions used in different countries, which could lead to temporal or spatial biases in case surveillance.”*

Line 556: “Locations with high baseline seroprevalence may experience few LF cases even if LASV is actively circulating in the rodent population.” This is an important point and while the subsequent impact of vaccinating a population in these contexts is beyond the scope of this manuscript it highlights that rodent pathogen dynamics should be included in the introduction as it is an important component of the disease system.

*As suggested in your comments above on lines 113-116 of the Introduction, we have added a mention regarding the influence of rodent population dynamics on spillover rates from rodents to humans.*

Line 610-620: Current hotspots are commonly associated with specialist treatment centers, awareness of the disease, surveillance systems and available diagnostics. Much of the remaining endemic area does not have access to these and so it may remain premature to rule out a similar burden (~50 confirmed annual deaths at adm1 level, presumably single digit reported deaths at adm2) in non-traditional hotspots.

*We have updated this paragraph to acknowledge that undetected hotspots may exist outside of current surveillance systems:*

*“Our modeling framework did incorporate estimation of country specific LF case and death reporting probabilities, but the extent to which LF hotspots might exist in areas that haven’t reported any LF cases or deaths is unknown. Current hotspots are associated with the presence of LF surveillance systems and the locations of specialty diagnostic and treatment centers, suggesting that transmission may be occurring undetected where these systems are lacking. For example, high seroprevalence was recently observed in southern Mali, suggesting that there may be undocumented areas of LF incidence outside of the historical hotspots.^85,86^ Although the burden of LF outside of the known hotspots is an important outstanding question, there is some evidence that observed spatial patterns of reported LF cases and deaths reflect at least some important differences in the spatial distribution of the disease.”*

Code review:
I ran the code for admin level 2 and a reversion rate of 6.

Update readme or script step1 to explain requirement to specify the level of analysis admin1/2 and seroreversion rate.

This code produced an error for me.
for(ii in 1:nrow(s)){
a[ii,] = a.tmp[which(paste(a.tmp$ADM1_code,a.tmp$YEAR) ==
paste(s$ADM1_code,pmin(2014,s$YEAR))[ii]),] }

Error in x[[jj]][iseq] <- vjj : replacement has length zero
I achieved the intended outcome with a <- merge(s, a.tmp, by.x = c("ADM1_code", "YEAR_adj"), by.y = c("ADM1_code", "YEAR"), all.x = TRUE)

*We fixed this error, which was caused by data from outside the study region. The reviewer’s code is a better way to achieve the same result.*

Update readme or comment line 20 in step2 to direct the user to extract data/adm_2_pop_upd.csv

*We have added this comment.*

step2_estimate_… line 72 priors have not been uploaded to the repo and it is unclear the structure they take so I have been unable to run the remaining script as these are necessary parameters for the subsequent functions.

*We have added the script that creates these priors.*

step3_ unable to run as required results from step_2. The code looks like it performs the expected analysis.
Unable to run step4_ beyond line 46
Haven’t run the remaining step4_/step5_ or step6_ scripts and challenging to check them based on code as written.

Overall, this is an important and timely manuscript that makes a valuable contribution to the field. Thank you for the opportunity to review it.

[NOTE: If reviewer comments were submitted as an attachment file, they will be attached to this email and accessible via the submission site. Please log into your account, locate the manuscript record, and check for the action link "View Attachments". If this link does not appear, there are no attachment files.]

**Figure resubmission:**

While revising your submission, please upload your figure files to the Preflight Analysis and Conversion Engine (PACE) digital diagnostic tool, [https://pacev2.apexcovantage.com/](https://gcc02.safelinks.protection.outlook.com/?url=https%3A%2F%2Fpacev2.apexcovantage.com%2F&data=05%7C02%7Csean.moore2%40nih.gov%7C312a86d2203b4e5acc3708dd407ae923%7C14b77578977342d58507251ca2dc2b06%7C0%7C0%7C638737619280806148%7CUnknown%7CTWFpbGZsb3d8eyJFbXB0eU1hcGkiOnRydWUsIlYiOiIwLjAuMDAwMCIsIlAiOiJXaW4zMiIsIkFOIjoiTWFpbCIsIldUIjoyfQ%3D%3D%7C0%7C%7C%7C&sdata=K4CC0c6u03Fy2c7LtLgQ1bhqjA5%2BUnkbe8SQoCslMrs%3D&reserved=0). PACE helps ensure that figures meet PLOS requirements. To use PACE, you must first register as a user. Registration is free. Then, login and navigate to the UPLOAD tab, where you will find detailed instructions on how to use the tool. If you encounter any issues or have any questions when using PACE, please email PLOS at [figures@plos.org](mailto:figures@plos.org). Please note that Supporting Information files do not need this step. If there are other versions of figure files still present in your submission file inventory at resubmission, please replace them with the PACE-processed versions.
